# The timing for initiating estrogen stimulation in artificial cycle for frozen-thawed embryo transfer can be flexible

**DOI:** 10.1186/s12978-021-01229-1

**Published:** 2021-09-09

**Authors:** Ying Ying, Yixuan Wu, Shuang Liu, Qing Huang, Haiying Liu

**Affiliations:** Department of Obstetrics and Gynecology, Center for Reproductive Medicine, Key Laboratory for Major Obstetric Diseases of Guangdong Province, The Third Affiliated Hospital of Guangzhou Medical University, 63 Duobao Road, Liwan District, Guangzhou, China

**Keywords:** Estrogen stimulation, Artificial cycle, Frozen-thawed embryo transfer

## Abstract

**Background:**

There remains a lack of evidence to demonstrate whether the initiation time of estrogen stimulation is flexible in the proliferative endometrial phase during the artificial cycle for frozen-thawed embryo transfer (AC-FET).

**Methods:**

FET records were retrospectively reviewed from a large university-affiliated reproductive medicine center. Only the patients who were undergoing their first embryo transfer with a single blastocyst in the AC-FET cycles were included: thereby 660 cycles were recruited, and the patients were grouped according to their day of estrogen usage initiation as early initiation group (estrogen stimulation initiated during days 2–5 of menses, n = 128) and the late initiation group (estrogen stimulation initiated on or after the 6th day of menses, n = 532). The primary outcome was the ongoing pregnancy rates (OPR).

**Results:**

The rates of biochemical and clinical pregnancies were significantly higher in the late initiation group relative to those in the early initiation group, however, no significant differences were noted between the two groups for OPR. Furthermore, after adjusting for the results of the potential confounders, no impact was observed in the initiation time of estrogen stimulation on the OPR.

**Conclusions:**

This study provides evidence that initiating the estrogen stimulation on after days 2–5 of menses do not exert adverse effects on the OPR in AC-FETs. Thus, AC-FET can be scheduled in a flexible manner without compromising on the pregnancy outcomes.

## Plain Language summary

Despite the continuous efforts invested in exploring and optimizing therapeutic regimens to improve the success rate of frozen-thawed embryo transfer (FET), we found that there are little evidence to demonstrate whether the initiation time of estrogen stimulation is flexible in the proliferative endometrial phase during the artificial cycle for FET (AC-FET). Thus, we retrospectively reviewed the FET records from a large university-affiliated reproductive medicine center to explore whether the relatively late start of endometrial stimulation for FET influences the pregnancy outcome. Results provided evidences that initiating the estrogen stimulation on after days 2–5 of menses do not exert adverse effects on the ongoing pregnancy rates in AC-FETs. Therefore, FET can be scheduled in a flexible manner, according to the ovulatory and endometrial statuses and patient and/or clinic preference, without compromised clinical outcomes.

## Background

Of late, the universal use of frozen-thawed embryo transfer (FET) in clinical practice has resulted in ideal pregnancy outcomes. Despite the dramatic rise in FET cycles over the recent years, there is insufficient evidence to recommend one protocol for endometrial preparation over another [1, 2], and customized approaches are needed [3]. Artificial cycle FET (AC-FET) prepares the endometrium by the administration of exogenous estrogen and progesterone. The former promotes endometrial proliferation and induces progesterone receptors, while the latter alters the physiological state of the endometrium from the proliferation phase to the secretion phase. The series of hormonal changes make the endometrium receptive [4, 5]. AC-FET mimics this physiological process.

To continuously improve the success rate of FET, several studies have attempted to optimize the therapeutic regimen by ascertaining the best doses and routes of administration for both estrogen and progesterone. Furthermore, efforts have been made to determine the optimum duration of estrogen administration prior to initiating progesterone administration. However, in clinical practice, different centers and even different doctors in the same center may show variations in the treatment protocols based on their individual concepts, experiences, and habits. For example, we know that when the pituitary is not suppressed using a gonadotropin releasing hormone agonist (GnRH-a), it is very important to start estrogen treatment in the early follicular phase (on day 1 or day 2), especially for those who ovulate regularly, because starting estrogen treatment after day 3 of the cycle might lead to an increased incidence of luteinizing hormone (LH) surge and the resultant luteinization of the endometrium [6]. However, we often encounter patients who come for consultation on the 6th day of their menstrual cycle or later and request endometrial preparation for FET. In general, such patients are those who had just completed oocyte retrieval and embryo cryopreservation, and they were instructed to consult the doctor on 3–5 days after the first menstruation is over, because they had to complete the leucorrhea examination before FET. If transvaginal ultrasound indicates no dominant follicle development in the bilateral ovaries, endometrial thickness is < 7 mm, and serum sex hormones are at basal levels, in addition to the patients’ time cost and personal willingness, the physician usually agrees to initiate the endometrial preparation program of AC-FET.

Therefore, we wished to ascertain whether the relatively late start of endometrial stimulation for FET influences the pregnancy outcome. Owing to the lack of a uniform standard, we defined “the late initiation of estrogen stimulation” as the onset of estrogen stimulation on or after the 6th day of natural menses or withdrawal bleeding. Thus, the present study was designed to assess whether the initiation time of estrogen stimulation in AC-FET cycles influences the pregnancy outcomes in patients undergoing their first cycle of vitrified-warmed, single blastocyst transfer.

## Methods

### Study design

This retrospective study was conducted at the Reproductive Medicine Center of The Third Affiliated Hospital of Guangzhou Medical University from January to December 2019. All FETs performed at our center during this period were reviewed for their potential inclusion in the study. Only patients who underwent their first embryo transfer after autologous in vitro fertilization (IVF) and single blastocyst thawing and transfer were included in this analysis. We identified AC-FETs without GnRH-a pituitary downregulation and included the records of women < 45 years of age at the time of FET. Since all patients underwent their first embryo transfer after IVF, all embryos were warmed and transferred within a year of vitrification. We excluded patients with preimplantation genetic testing (PGT) cycles, those with peak endometrial thickness of < 6 mm, those with embryos derived from vitrified oocytes, and those with incomplete records. The study protocol was approved by the Ethical Committee of The Third Affiliated Hospital, Guangzhou Medical University.

### Frozen embryo transfer protocol

Controlled ovarian stimulation, trigger injection, oocyte retrieval, embryo culture, embryo transfer, and cryopreservation were conducted according to the standard protocols. Fertilization was achieved via conventional IVF or intracytoplasmic sperm injection depending on the semen parameters and the history of prior IVF outcomes. Fresh embryo transfers were conducted either on Day 3 or Day 5 based on the embryo quality, number, and clinical indications. Similarly, the embryos were vitrified either in the cleavage or the blastocyst stage, depending on their quality, number, and clinical indications.

FET was performed through a programmed or natural cycle as per the doctor’s experience and the patient’s specific situation and requirements. Only those women undergoing AC-FET without GnRH-a downregulation were included in this study. On days 2–5 of spontaneous menses or withdrawal bleeding, the patients underwent a baseline transvaginal ultrasound (TVUS) as well as an assessment of serum luteinizing hormone, estrogen, and progesterone to confirm that they were in the early proliferative phase of their menstrual cycle. In patients reporting late for various reasons as mentioned earlier, these investigations were performed on Day 6 of their menstrual cycle or beyond. Therapy was initiated with oral estrogen (estradiol valerate, Progynova, Bayer), 3 mg twice daily. TVUS was performed within 7–10 days of initiating estrogen supplementation to assess the recipients’ endometrium before adjusting the estrogen doses. Serum progesterone was measured at each visit to rule out premature ovulation before the initiation of progesterone supplementation. After at least 7 days of initiating estrogen administration, vaginal micronized progesterone supplementation (200 mg, three times per day) was started if the endometrial thickness was > 7 mm. The transfer of frozen-thawed blastocysts was performed after 5 days of progesterone supplementation.

### Embryo grading

The blastocysts were graded according to the following three morphological parameters: inner cell mass (ICM), trophectoderm, and the degree of expansion with a hatching stage [7]. At our center, blastocysts of grade ≥ 4BB were defined as high-quality blastocysts. Vitrification of the blastocysts was performed on days 5 or 6 based on the development of each embryo.

### Main outcome measures and statistical analyses

The primary outcome measure was ongoing pregnancy, which was defined as the visualization of fetal cardiac activity on TVUS at ≥ 12 weeks of gestation. The secondary outcomes included biochemical pregnancy (defined as cases with increased serum human chorionic gonadotropin levels 14 days after embryo transfer) and clinical pregnancy (confirmed by ultrasonographic visualization of a gestational sac 4–5 weeks after embryo transfer).

The continuous data were expressed as median (lower and upper quartiles), and the categorical data were expressed as counts (percentages). The parameters of the different groups were initially assessed using Mann–Whitney U-test for continuous variables and Chi-square test for categorical variables. Odds ratios (OR) with 95% confidence intervals (CI) were calculated and adjusted for patient’s age, body mass index (BMI), duration of estrogen usage, peak endometrial thickness, optimal embryos transferred, and initial day of estrogen administration using both univariable and multivariable logistic regression. All tests were two-sided and were performed using IBM Statistical Package for the Social Sciences software package, version 20. p < 0.05 was considered statistically significant.

## Results

**Fig. 1:**
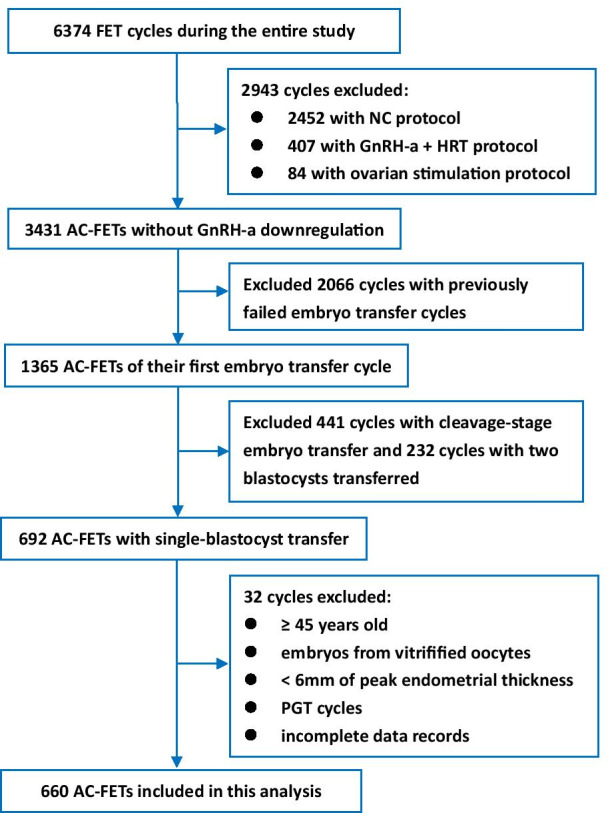
Flowchart depicting the eligibility criteria for patient inclusion

During the study period, a total of 6374 FET cycles were performed at our center. Among these, 660 cycles of AC-FETs involving first embryo transfer (single-blastocyst transfer) were included in the present analysis. (Fig. 1).

Patients were divided into two groups according to the initial day of estrogen usage, as follows: early initiation group (estrogen stimulation started from days 2–5 of spontaneous menstruation or withdrawal bleeding, n = 128) and late initiation group (estrogen stimulation commenced on or after the 6th day of spontaneous menstruation or withdrawal bleeding, n = 532). The demographic characteristics and the FET parameters of the patients are summarized in Table 1. No significant differences were noted between the two groups in terms of the patients’ age, BMI, infertility type and duration, infertility factor, and type of menstruation. However, the duration of estrogen usage was significantly longer in the early initiation group (14 days [11, 16]) than in the late initiation group (11 days [9, 14]). Besides, the peak endometrial thickness was significantly thicker in the late initiation group (8.4 mm [7.8, 9.5]) when compared with the early initiation group (8.0 mm [7.4, 9.5]). Nonetheless, no significant differences were noted between the two groups in the endometrial thickness on the 1st day of estrogen usage and the percentages of optimal embryos transferred. The rates of biochemical and clinical pregnancies were significantly higher in the late initiation group than in the early initiation group (68.0% versus 54.7% and 64.3% versus 51.6%, respectively). However, no significant differences were observed between the two groups in the ongoing pregnancy rate.

**Table 1:** Demographic characteristics and FET parameters of the study population according to the initial day of estrogen usage

Parameters	Early initiation group (n = 128)	Late initiation group (n = 532)	*p* value
Age, years	30 (28, 34)	30 (27, 32)	0.063
BMI, kg/m^2^	21.8 (20.1, 24.6)	22.0 (20.0, 24.5)	0.830
Infertility type, n (%)			0.486
Primary	77 (60.2)	302 (56.8)	
Secondary	51 (39.8)	230 (43.2)	
Infertility duration, years	3 (2, 5)	4 (2, 5)	0.228
Infertility factor, n (%)			0.061
Tubal factor	69 (53.9)	219 (41.2)	
Male factor	19 (14.8)	93 (17.5)	
Mixed male and female factor	33 (25.8)	191 (35.9)	
Unexplained	7 (5.5)	29 (5.5)	
Menstruation, n (%)			0.993
Regular	69 (53.9)	287 (53.9)	
Irregular	59 (46.1)	245 (46.1)	
Duration of estrogen usage, days	14 (11, 16)	11 (9, 14)	**< 0.001**
Endometrial thickness at initial day of estrogen usage, mm	5.0 (4.1, 5.8)	5.9 (4.0, 5.7)	0.469
Peak endometrial thickness, mm	8.0 (7.4, 9.5)	8.4 (7.8, 9.5)	**0.037**
Optimal embryos transferred, n (%)	107 (83.6)	465 (87.4)	0.255
Biochemical pregnancy, n (%)	70 (54.7)	362 (68.0)	**0.004**
Clinical pregnancy, n (%)	66 (51.6)	342 (64.3)	**0.008**
Ongoing pregnancy, n (%)	60 (46.9)	290 (54.5)	0.120

The continuous data were expressed as **median (lower and upper quartiles)**

Moreover, patients were divided into two groups according to the presence or absence of ongoing pregnancy, as follows: ongoing pregnancy group (n = 350) and no ongoing pregnancy group (n = 310). The demographic characteristics and FET parameters of the patients are summarized in Table 2.

**Table 2:** Demographic characteristics and FET parameters of the study population according to ongoing pregnancy

Parameters	Ongoing pregnancy (n = 350)	No ongoing pregnancy (n = 310)	*p* value
Age, years	29 (27, 32)	30 (28, 34)	**0.001**
BMI, kg/m^2^	21.8 (20.1, 24.0)	22.2 (20.0, 25.0)	0.113
Infertility type, n (%)			0.751
Primary	203 (58.0)	176 (56.8)	
Secondary	147 (42.0)	134 (43.2)	
Infertility duration, years	3.5 (2, 5)	3 (2, 5)	0.549
Infertility factor, n (%)			0.349
Tubal factor	164 (46.9)	124 (40.0)	
Male factor	55 (15.7)	57 (18.4)	
Mixed male and female factor	112 (32.0)	112 (36.1)	
Unexplained	19 (5.4)	17 (5.5)	
Menstruation, n (%)			0.902
Regular	188 (53.7)	168 (54.2)	
Irregular	162 (46.3)	142 (45.8)	
Initial day of estrogen usage	11 (8, 13)	10 (7, 13)	0.387
Duration of estrogen usage, days	11 (10, 15)	11 (9, 15)	0.331
Endometrial thickness at initial day of estrogen, mm	5.0 (4.0, 5.7)	4.8 (4.1, 5.8)	0.879
Peak endometrial thickness, mm	8.5 (7.8, 9.6)	8.2 (7.6, 9.4)	0.076
Optimal embryos transferred, n (%)	321 (91.7)	251 (81.0)	**< 0.001**

The continuous data were expressed as **median (lower and upper quartiles)**

The patients in the ongoing pregnancy group were significantly younger than those in the no ongoing pregnancy group. Nevertheless, no significant differences were observed between the two groups in terms of BMI, infertility type and duration, infertility factor, and type of menstruation. Furthermore, the initial day and duration of estrogen usage, endometrial thickness on the 1st day of estrogen administration, and peak endometrial thickness were not significantly different between the two groups. The percentages of optimal embryos transferred were significantly higher in the ongoing pregnancy group than in the no ongoing pregnancy group (91.7% versus 81.0%, p < 0.001).

In the multivariable logistic regression model (Table 3), after adjusting the results for the potential confounders, including women’s age, BMI, initial day of estrogen usage, duration of estrogen usage, peak endometrial thickness, and optimal embryos transferred, only the women’s age (OR 0.942, 95% CI 0.904–0.982, p = 0.005) and morphologically optimal embryos transferred (OR 2.321, 95% CI 1.429–3.770, p = 0.001) were inferred to be important independent prognostic factors for confirming an ongoing pregnancy. Thus, the results suggested that the initial time of estrogen stimulation should not be considered as a prognostic factor for ongoing pregnancy.

**Table 3:** Univariable and multivariable logistic regression analysis of factors related to ongoing pregnancy

Variables	Univariable analysis		Multivariable analysis	
	OR (95% CI)	*p value*	OR (95% CI)	*p value*
Age	0.928 (0.892–0.966)	**< 0.001**	0.942 (0.904–0.982)	**0.005**
BMI	0.961 (0.920–1.005)	0.083	0.965 (0.922–1.010)	0.129
Initial day of estrogen usage	1.016 (0.978–1.054)	0.420	1.021 (0.981–1.063)	0.308
Duration of estrogen usage	1.018 (0.979–1.059)	0.371	1.037 (0.992–1.084)	0.109
Peak endometrial thickness	1.081 (0.969–1.205)	0.162	1.115 (0.991–1.255)	0.070
Optimal embryos transferred	2.602 (1.619–4.180)	**< 0.001**	2.321 (1.429–3.770)	**0.001**

## Discussion

To date, many studies have explored ways to optimize AC-FET in various aspects to make the endometrium receptive, including the selection of the best doses and routes of administration for both estrogen and progesterone as well as the optimum duration of estrogen administration prior to initiating progesterone.

To the best of our knowledge, this is the first study to determine whether the initial time of estrogen stimulation influences the ongoing pregnancy in AC-FET. At the very beginning, downregulation of GnRH-a was important before estrogen stimulation in AC-FET cycles. In the later stage, it turned out that the LH rise and endometrium luteinization rarely occurred without GnRH-a downregulation. However, if the pituitary is not suppressed using a GnRH-a, it is very important to start estrogen treatment in the early follicular phase (on Day 1 or Day 2), especially for those with normal ovulation. Therefore, estrogen stimulation is routinely started on days 2–5 of menstruation or withdrawal bleeding in our center. Nonetheless, many patients do start hormone treatment beyond this window owing to various reasons, and this happens quite often in our center. We did not find that this delay has an impact on the overall success rate.

Our data showed that patients with late estrogen initiation (on or after the 6th day of natural menses or withdrawal bleeding) shared similar ongoing pregnancy rates as those who initiated the treatment within the normal time range. Significant differences were however noted in the duration of estrogen usage and the peak endometrial thickness between the two groups. After adjusting the results for the potential confounders, including the above-mentioned parameters, it was evident the initial time of estrogen stimulation should not be viewed as an important independent prognostic factor for confirming ongoing pregnancy.

A duration of 10–14 days of estrogen administration is routinely utilized in artificial protocols; however, for those patients who do not respond within this time, the duration should be extended. Histological evaluations have revealed that 5 weeks of estrogen priming is ideal for obtaining optimal luteal phase endometrial histology after progesterone treatment [8]. Positive results have been reported after prolonged (4–5 weeks) vaginal estrogen administration [9]. Therefore, there does not appear to be an upper limit for the duration of estrogen stimulation. However, one study has reported that prolonged unopposed estrogen administration beyond 40 days is associated with a high rate of breakthrough bleeding [10]. In our study, the longest duration of estrogen administration was 26 days, and no breakthrough bleeding was observed in the study population.

When the patients were divided into ongoing pregnancy and no ongoing pregnancy groups, no significant difference was found in the initial day of estrogen usage between the two groups. The patients were significantly younger and the percentages of optimal embryos transferred were significantly higher in the ongoing pregnancy group than in the other group. However, after adjusting the results for the potential confounders using multivariate logistic regression analysis, the results signified that the initial time of estrogen stimulation should not be considered as a prognostic factor for ongoing pregnancy.

The strength of our study lies in our inclusion of the impacts of many factors such as the women’s age, previous failed embryo transfer cycles, embryo development stage, number of embryos transferred, and endometrial thicknesses. Despite our cautions, our study has certain limitations. The first one is related to the inevitable bias introduced by the retrospective nature of the study. Second, some baseline characteristics differed between the groups, after adjusting for the confounders, we still observed that the initial time of estrogen stimulation did not have an impact on the ongoing pregnancy rate. Finally, we did not analyze the live birth rates because of the non-availability of complete patients records. Despite these limitations, our study provides valuable data for the physicians employing the flexible artificial protocol.

## Conclusions

This study provides evidence that initiating estrogen stimulation beyond the window of 2–5 days of menses or withdrawal bleeding does not adversely affect the ongoing pregnancy. Our findings imply that AC-FET can be scheduled in a flexible manner without compromising the pregnancy outcome. However, well-designed, prospective clinical trials are needed to further assess the effects of late initiation of estrogen administration in AC-FET.

## Data Availability

The datasets used and/or analyzed during the current study are available from the corresponding author on reasonable request.

## Abbreviations

AC-FET: Artificial cycle for frozen-thawed embryo transfer
OPR: Ongoing pregnancy rates
GnRH-a: Gonadotropin releasing hormone agonist
LH: Luteinizing hormone
IVF: In vitro fertilization
PGT: Preimplantation genetic testing
TVUS: Transvaginal ultrasound
